# Integration of Neighbor Topologies Based on Meta-Paths and Node Attributes for Predicting Drug-Related Diseases

**DOI:** 10.3390/ijms23073870

**Published:** 2022-03-31

**Authors:** Ping Xuan, Zixuan Lu, Tiangang Zhang, Yong Liu, Toshiya Nakaguchi

**Affiliations:** 1 School of Computer Science and Technology, Heilongjiang University, Harbin 150080, China; xuanping@hlju.edu.cn (P.X.); zxuanlu2270@163.com (Z.L.); 2 School of Mathematical Science, Heilongjiang University, Harbin 150080, China; 3 Center for Frontier Medical Engineering, Chiba University, Chiba 2638522, Japan; nakaguchi.chibau@gmail.com

**Keywords:** drug–disease association prediction, neighbor topology learning based on meta-paths, pairwise node attribute encoding, multiple drug–disease heterogeneous networks, fully connected neural networks and autoencoder based on CNN

## Abstract

Identifying new disease indications for existing drugs can help facilitate drug development and reduce development cost. The previous drug–disease association prediction methods focused on data about drugs and diseases from multiple sources. However, they did not deeply integrate the neighbor topological information of drug and disease nodes from various meta-path perspectives. We propose a prediction method called NAPred to encode and integrate meta-path-level neighbor topologies, multiple kinds of drug attributes, and drug-related and disease-related similarities and associations. The multiple kinds of similarities between drugs reflect the degrees of similarity between two drugs from different perspectives. Therefore, we constructed three drug–disease heterogeneous networks according to these drug similarities, respectively. A learning framework based on fully connected neural networks and a convolutional neural network with an attention mechanism is proposed to learn information of the neighbor nodes of a pair of drug and disease nodes. The multiple neighbor sets composed of different kinds of nodes were formed respectively based on meta-paths with different semantics and different scales. We established the attention mechanisms at the neighbor-scale level and at the neighbor topology level to learn enhanced neighbor feature representations and enhanced neighbor topological representations. A convolutional-autoencoder-based module is proposed to encode the attributes of the drug–disease pair in three heterogeneous networks. Extensive experimental results indicated that NAPred outperformed several state-of-the-art methods for drug–disease association prediction, and the improved recall rates demonstrated that NAPred was able to retrieve more actual drug–disease associations from the top-ranked candidates. Case studies on five drugs further demonstrated the ability of NAPred to identify potential drug-related disease candidates.

## 1. Introduction

The process of producing a new medicine is typically lengthy, expensive, and fraught with failure; it may require more than 10 y and cost between USD 0.8 billion and USD 1.5 billion on average [1,2,3,4,5]. Therefore, a method to reduce the time and funding costs for the development of new medicines must be identified. That approved drugs are subject to clinical trials endows them with a favorable safety profile. In contrast to developing a medicine from scratch, using indications for current drugs (drug repositioning) [6] can effectively reduce research and development costs and accelerate drug development [7,8,9].

Drug candidates can be further screened for wet laboratory validation using computational predictions of the relationship between licensed drugs and diseases [10,11]. Several approaches for predicting drug-related diseases that have been reported can be classified into two categories. The first category of methods predicts the disease indications for drugs based on the integration of multiple kinds of information about the drugs and diseases. A couple of methods integrate the known drug–disease associations, the drug similarities, and the disease similarities [12,13]. They estimate the association possibilities between drugs and diseases by utilizing a logistic regression classifier and matrix decomposition with a similarity constraint. Wang et al. employed kernel functions to incorporate drug and disease similarities and applied the support vector machine approach to forecast drug–disease correlations [14]. Liang et al. applied sparse subspace learning and graph Laplacian regularization to combine multiple types of drug characteristics to predict drug indications [15]. To infer drug–disease associations, relevant data from drugs and diseases are utilized or combined in these strategies. However, the above-mentioned approaches cannot consider topological information in a network to demonstrate the potential use of a specific drug.

The second method primarily considers prediction based on the topology of the network. For example, heterogeneous network models based on diseases, drugs, and targets are used to infer drug candidates using iterative algorithms [16]. In several methods, random walk algorithms are employed to predict possible drug–disease associations; in fact, they have been employed in networks such as drug similarity, disease similarity, and integrated drug–disease heterogeneity networks [17,18,19,20,21]. However, because these methods do not consider the attribute information of drug and disease network nodes, they cannot learn the deep feature representation of nodes. Furthermore, these shallow-model-based approaches cannot extract potentially complicated relationships between drug and disease nodes.

Deep learning technologies have been widely utilized for the prediction of miRNA–disease associations [22] and disease-related lncRNAs [23,24]. Owing to the development of deep learning, the indications of drug candidates are identified more accurately in recent approaches by integrating multiple sources of drug- and disease-relevant information. For the prediction of drug-related diseases, models employing graph convolutional and fully connected autoencoders with attention mechanisms are used [25]. Xuan et al. [26] proposed a prediction model comprising a convolutional neural network (CNN) and a bi-directional long short-term memory (BiLSTM) network. Jiang et al. devised a module for forecasting drug–disease correlations by employing Gaussian interaction profile kernels and autoencoders [27]. Deep relationships between drugs and diseases can be extracted more easily using deep learning models. At the node pair level, however, the present deep learning approaches cannot combine and incorporate the drug–disease neighbor topology and attribute information. In addition, when capturing the neighbor topology information in three heterogeneous networks, the multi-scale meta-paths to obtain the set of neighbor nodes is important auxiliary information.

Herein, we propose and develop NAPred, a predictive model for capturing, encoding, and learning the neighbor topology and attribute representation of node pairs from diverse heterogeneous networks. The primary contributions of our proposed model are as follows:

- Three drug–disease heterogeneous networks were constructed, each with different aspects of drug similarities, to facilitate the acquisition of topological information regarding drug and disease nodes from different perspectives. To construct sets of different types of neighbors of the nodes, multi-scale meta-path sets of drug or disease nodes were established;
- We present an approach based on fully connected and convolutional neural networks with attention mechanisms for learning topological information regarding the same type of neighbors for drug and disease nodes. Multiple-neighbor feature representations extracted from drug and disease nodes were adaptively combined via a neighbor-scale-level attention mechanism;
- We developed a neighbor-topology-level attention mechanism to distinguish the contributions and then obtain the neighbor topological representations of the nodes; this is because different types of neighbor topological features contribute differently to drug–disease association prediction;
- The attribute information of the node pairs was extracted from the three heterogeneous networks using the proposed embedding mechanism and encoded using a convolutional autoencoder (CAE). The premise of this embedding mechanism is that drug–disease pairs are more likely to be associated with each other if they exhibit similarities or associations with more typical drugs or diseases.

## 2. Experimental Results and Discussion

### 2.1. Evaluation Metrics

The performances of all prediction models were analyzed and compared using five-fold cross-validation. Positive and negative samples were those with known and unknown drug–disease associations, respectively. We used 4/5 of the positive samples, as well as 4/5 of the random negative samples formed in the training set in each fold of the cross-validation. The remaining 1/5 positive samples, as well as all negative samples were tested. The prediction correlation scores of the test samples were generated and ranked; the higher the rank of the positive sample use cases, the better was their prediction performance.

Several evaluation metrics were used in this study, i.e., the true positive rate (TPR), false positive rate (FPR), receiver operating characteristic (ROC) curve, area under the ROC curve (AUC) [28], precision–recall (PR) curve, area under the PR (AUPR) curve [29], and recall at various top-*k*. The performances of all models in the cross-validation were compared based on the average AUC and AUPR.

The AUC is an accepted appraisal metric for comparing algorithms and probabilistic estimates [30]. The TPR and FPR at various thresholds yield the ROC curve. The sample was regarded as positive if the predicted association score of a drug–disease pair exceeds a threshold θ; otherwise, it was considered negative. The fraction of correctly (incorrectly) detected positive (negative) samples among all the positive (negative) samples is denoted as the TPR (FPR).

(1) $$TPR=\frac{TP}{TP+FN},FPR=\frac{FP}{TN+FP},$$

where TP (FN) represents the number of positive samples correctly (incorrectly) classified as positive (negative) and TN (FP) indicates the number of negative samples correctly (incorrectly) categorized as negative (positive) [31,32].

This was due to the uneven distribution of drug–disease candidates. The AUPR curve provides more information regarding the AUC for assessing the predictive performance [29]. precision and recall were determined as follows:

(2) $$precision=\frac{TP}{TP+FP},recall=\frac{TP}{TP+FN}$$

where precision indicates the rate of TP samples among those anticipated to be positive and recall expresses the rate of positive samples accurately recognized among the total positive samples. The AUC and AUPR curve were calculated using the mean cross-validation [33]. Each fold’s mean AUC and AUPR curve must be calculated, and the final score is the average of the five results.

Considering that biologists typically choose the top-ranked candidates and confirm their predictions based on wet laboratory trials, determining the actual drug–disease connections is critical. Therefore, for the projected outcomes, the recall rates of the top-*k* candidate drug–disease pairs were evaluated. The more trustworthy the prediction performance, the higher is the recall of the top-*k*.

### 2.2. Comparison with Other Methods

NAPred is more effective compared with six cutting-edge drug–disease association forecasting models: GFPred [25], CBPred [26], SCMFDD [13], LRSSL [15], MBiRW [18], and HGBI [16]. In the cross-validation, the other six methods were trained or tested using the same or similar datasets as the NAPred model. The best performance was achieved by each method when the optimal parameters were used. In particular, lr=0.001 for GFPred; lr=0.001 and λ=0.12 for CBPred; μ=λ=0.01, γ=2, and k=10 for LRSSL; α=0.3, c=−11, d=log(9999), and l=r=2 for MBiRW; k=45%, µ=1, and λ=4 for SCMFDD; α=0.4 for HGBI.

**Figure 1:**
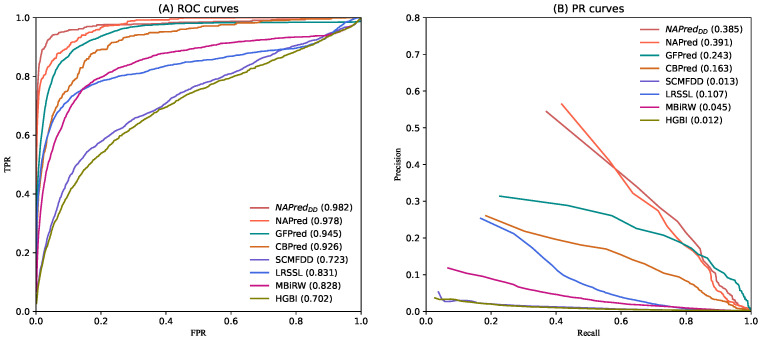
ROC and PR curves of all the methods of drug–disease association.

For each of the 763 drugs, we calculated the AUC and AUPR curve at each fold before calculating their five-fold mean. The final results were averaged across all AUCs (or AUPR curves) for the 763 drugs. As shown in Figure 1A, in the comparison of the 763 drugs, NAPred achieved the best mean AUC value among all the methods investigated (AUC = 0.978), outperforming GFPred by 3.3%, CBPred by 5.2%, SCMFDD by 25.5%, LRSSL by 14.7%, MBiRW by 15%, and HGBI by 27.6%. The second-best model GFPred successfully learned multiple attribute representations of nodes and fully extracted topological information from multiple heterogeneous networks. This suggests that constructing heterogeneous networks on the basis of multiple drug similarities and capturing topological information improved the prediction accuracy. CBPred, LRSSL, and MBiRW extract topology information from heterogeneous networks for drug repositioning, where CBPred considers the path information between pairs of diseases, whereas MBiRW disregards the properties of the nodes. Hence, CBPred performed better, whereas MBiRW performed worse than LRSSL. SCMFDD is a matrix-decomposition-based model. The dimensionality reduction process may cause the lossof low-frequency valid information. Therefore, SCMFDD performed worse, but better than HGBI; additionally, it did not exploit the multiple similarities of the drugs. In conclusion, our NAPred achieved the best results owing to the comprehensive learning of the neighborhood topology, as well as the property information of the drug–disease pairs.

As shown in Figure 1B, our method NAPred performed better than GFPred, CBPred, LRSSL, MBiRW, SCMFDD, and HGBI by 14.8%, 22.8%, 28.4%, 34.6%, 37.8%, and 37.9%, respectively, based on the AUPR curves of 763 drugs.

In addition, to validate the robustness of our model under multiple datasets, we used the CC dataset [34] to replace drug-related data and implement another instance of our method, ${NAPred}_{DD}$. We utilized the A (chemistry) data, B (targets) data, and C (networks) data of CC dataset to replace the original chemical substructure, protein structural domain, and gene ontology data of the drugs. In Figure 1, the AUC and AUPR of ${NAPred}_{DD}$ are still higher than those of the compared methods. The experimental results demonstrated the good robustness of our model.

To evaluate the impact of cross-validation folds on NAPred performance, we also performed an additional ten-fold cross-validation. The number of training samples in the ten-fold cross-validation was larger than that in the five-fold cross-validation. As shown in Supplementary Table S1, the AUC and AUPR for the ten-fold cross validation were 0.8% and 1.3% higher than the five-fold cross validation. NAPred achieved better performance when the training data were increased.

The Wilcoxon test was used to evaluate the ability of the 763 drugs to predict the outcomes. NAPred performed much better than the other approaches in terms of the AUCs and AUPR curves when a 0.05 *p*-value threshold was used (Table 1).

**Figure 2:**
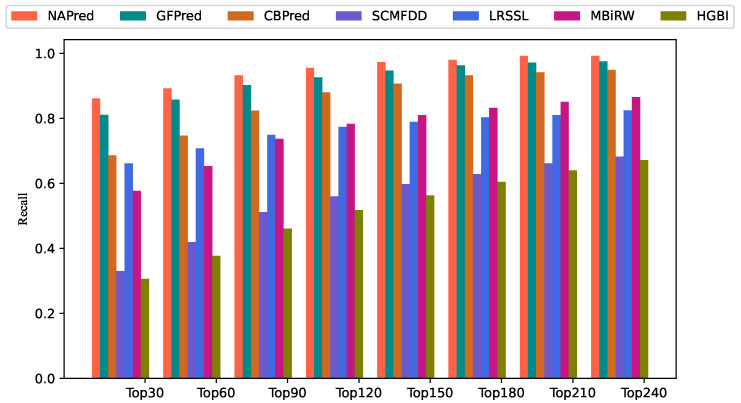
The average recalls of all the drugs under different top-*k*.

Figure 2 shows the recall rates of drug candidates for various top-*k* values. More real drug–disease associations can be successfully identified using a higher recall rate. The average recall rate for the 763 drugs was 86.14%, 89.19%, 93.24%, 95.54%, and 97.33% for the top-30, -60, -90, -120, and -150, respectively. Among the top-30, -90, -150, and -210, GFPred indicated the second-highest recall rate, with 81.03%, 90.20%, 94.64%, and 97.12%, respectively. CBPred obtained recall rates of 68.63%, 82.41%, 90.69%, and 94.17% in the top-30, -90, -150, and -210, respectively, with a slightly lower performance than GFPred. LRSSL demonstrated a higher recall than MBiRW for the top-30, -60, and -90. The former model achieved 66.12%, 70.73%, and 74.90% recall rates, whereas the latter obtained recall rates of 57.65%, 65.30%, and 73.71%, respectively. The recall of SCMFDD was 32.97%, 51.18%, 59.75%, and 66.13% when *k* was 30, 90, 150, and 210, respectively. HGBI had a slightly lower recall rate than SCMFDD, i.e., 30.62%, 46.10%, 56.34%, and 63.98% for the top-30, -90, -150, and -210, respectively.

### 2.3. Case Studies of Five Drugs

Case studies of ampicillin, ceftriaxone, doxorubicin, erythromycin, and itraconazole were conducted to further illustrate the efficacy of NAPred in drug–disease association prediction. The association prediction scores for each drug candidate in the descending order, as well as the top-ten candidates for each of the five drugs are listed in Table 2.

The Comparative Toxicogenomics Database (CTD), which was painstakingly acquired and validated based on the literature, contains information regarding drugs and their effects on human health [35]. DrugBank is a database containing drug-related targets, mechanisms of action, interactions, and integrated molecular information [36]. A total of 16 candidate diseases are covered by CTD, and 23 candidates are recorded in DrugBank. This indicates that the disease candidate was receiving effective treatment.

ClinicalTrials.gov, which is the world’s largest searchable clinical trial database, contains data pertaining to clinical studies conducted worldwide; the National Library of Medicine in the United States contributes to its resources. As Supporting Material, we only used experimental records with a “completed” status. PubChem is a public database sponsored by the National Institutes of Health that includes information regarding chemicals and their biological activity, safety, and toxicity [37]. There were 23 candidate diseases supported by ClinicalTrials.gov, whereas PubChem approved 33 of the candidates. These records indicate that clinical trials established an association between the candidate disease and the relevant drug.

Besides manually validated drug–disease correlations, CTD additionally includes those derived from the literature with temporarily unverified associations. The inferred section of the CTD contains two candidates, which suggests a more plausible correlation between the diseases and their corresponding drugs. Among all 50 drug candidates, two candidates were labeled as “unconfirmed”.

In addition, we conducted case studies on an additional five drugs (betamethasone, acetaminophen, etoposide, flurbiprofen, and verapamil) and list their top-ten candidate diseases in Supplementary Table S2. There were 42 candidate diseases recorded by CTD. There were 29 and 42 candidates covered by DrugBank and PubChem. ClinicalTrials contained 20 candidate diseases. This indicates that these candidates are more likely to be associated with the corresponding drugs. Only one candidate was labeled as “unconfirmed”. All the above analysis indicated that NAPred had the ability to discover potential candidate drug–disease associations.

### 2.4. Prediction of Novel Drug-Related Diseases

Finally, we applied the trained NAPred to 763 drugs to predict candidate diseases. The top-30 drug-related candidate diseases selected by our model are listed in Supplementary Table S3. They can be used by biologists to facilitate further wet experiments for validation.

## 3. Materials and Methods

**Figure 3:**
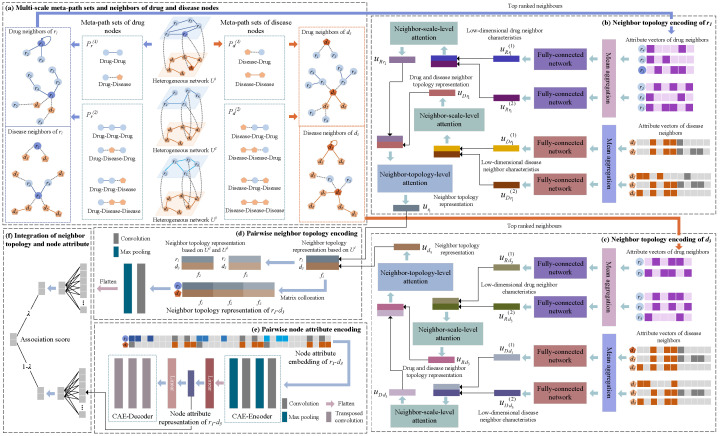
Framework of the proposed NAPred model. (**a**) Construct multi-scale meta-path sets and the sets composed of the same-type neighbor nodes. (**b**) Encode the attribute vectors of neighbor nodes of a drug. (**c**) Encode the attribute vectors of neighbor nodes of a disease. (**d**) Learn the neighbor topology of a drug–disease node pair. (**e**) Learn the attributes of the node pair. (**f**) Integrate multiple representations.

Figure 3 shows our proposed predictive model for drug-related disease candidates; the model comprises two branches. Three drug–disease heterogeneity networks were first established to correlate the similarities between drugs and diseases from different perspectives. For the first branch, we obtained the sets of neighbor nodes for drugs and diseases based on meta-paths of different scales. Neighbor-scale-level and neighbor-topology-level attention mechanisms are proposed for capturing drug and disease neighbor information, followed by encoding pairwise neighbor topology representations using convolutional neural networks. In the second branch, CAE was utilized to learn a pair of drug–disease attribute representations from the three drug–disease heterogeneous networks. The scores predicted from the two branches were weighted and summed to obtain the scores for the corresponding associations. A higher score signifies the higher possibility of an association.

### 3.1. Dataset

Based on previous studies, we obtained drug–disease association data [15], chemical substructure data of drugs, protein structural domain data of target proteins, and gene ontology information of target proteins. Initially, data pertaining to drug–disease associations were obtained in the UMLS [38], which contains information regarding 763 drugs, 681 diseases, and 3051 known drug–disease associations. We extracted drug chemical substructure data from the PubChem database [39] and drug target protein structural domain data from the InterPro database [40]. The UniProt database was used to obtain gene ontology information regarding the target protein of the drug [41]. The numbers of drug chemical substructures, drug target protein structural domains, and drug target protein gene ontologies in our dataset were 623, 1426, and 4447, respectively.

### 3.2. Establishing Drug–Disease Heterogeneous Networks

#### 3.2.1. Matrix of Drug Properties

Let the matrix $T^{c}$ denote the case in which each drug contains a chemical substructure, and $T^{c}\in R^{N_{r}\times N_{c}}$. $N_{r}$ and $N_{c}$ indicate the number of drugs and all relevant chemical substructures, respectively. A $T_{ij}^{c}$ value of 1 implies that drug $r_{i}$ contains the chemical substructure $c_{j}$, whereas a value of 0 implies otherwise. The vector of the chemical substructure attributes of $r_{i}$, which is obtained from the *i*-th row vector of $T^{c}$, is represented as $T_{i}^{c}$.

Let the matrix $T^{p}\in R^{N_{r}\times N_{p}}$ denote the cases of protein structural domains discovered in the respective associated target proteins of $N_{r}$ drugs; subsequently, $N_{p}$ is the number of protein structural domains of all drug target proteins. $T_{ij}^{p}$ is 1 for the target protein related to drug $r_{i}$ containing the *j*-th protein structural, and 0 otherwise. The protein structural domain attribute vector of $r_{i}$ is obtained from the *i*-th row of data in $T^{p}$.

The matrix $T^{g}\in R^{N_{r}\times N_{g}}$ is used to indicate whether $N_{g}$ gene ontology information is included in $N_{r}$ drugs and their associated target proteins. A $T_{ij}^{g}$ value of 1 implies that the target protein associated with drug $r_{i}$ contains gene ontology $g_{j}$, whereas a value of 0 implies otherwise. The target protein gene ontology property vector of $r_{i}$ is represented by the *i*-th row vector $T_{i}^{g}$.

#### 3.2.2. Establishment of the Drug Network

For two drugs $r_{i}$ and $r_{j}$, a higher number of identical chemical substructures between them signifies a higher level of similarity between them. The cosine similarity of their chemical substructures can be calculated using the strategy previously described by Liang et al. [15]; in fact, we used it as the first cosine similarity between $r_{i}$ and $r_{j}$.

Similarly, based on the protein domains or protein-associated gene ontologies in the two drug-related target proteins, cosine similarity calculations can be applied to determine the second and third similarities of a drug.

**Figure 4:**
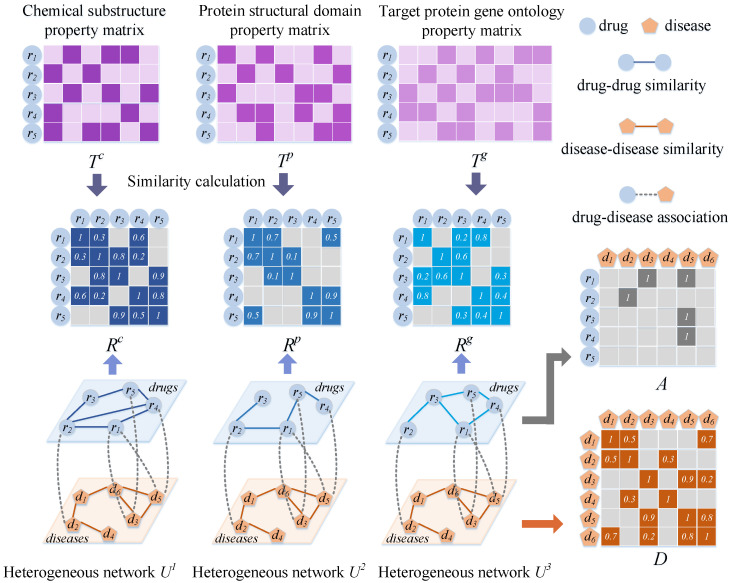
Construction of three heterogeneous networks based on multiple kinds of drug similarities, drug–disease associations, and disease similarities.

We treated two drug nodes as having connected edges when the calculated drug similarity exceeded 0. The weights on the edges are expressed as the similarity between the two drugs (Figure 4). We used the matrices $R^{c}=\left[\begin{array}{c} {R^{c}}_{ij} \end{array}\right]\in R^{N_{r}\times N_{r}}$, $R^{p}=\left[\begin{array}{c} {R^{p}}_{ij} \end{array}\right]\in R^{N_{r}\times N_{r}}$, and $R^{g}=\left[\begin{array}{c} {R^{g}}_{ij} \end{array}\right]\in R^{N_{r}\times N_{r}}$ to denote the drug networks obtained based on the similarity of the three drugs. For instance, based on the chemical substructure, $R_{ij}^{c}$ represents the similarity between $r_{i}$ and $r_{j}$.

#### 3.2.3. Establishment of the Disease Network

The similarity of diseases was calculated to establish disease networks. Wang et al. [42] computed the similarity between diseases using their directed acyclic graph (DAG). A DAG that includes all semantic terms associated with a disease can be used to illustrate the disease. A higher number of disease terms in the DAGs of two diseases implies a higher semantic similarity between them. The corresponding edges between any two diseases can be added if their similarity exceeds 0. The weights on these edges reflect the similarity between the two diseases. The matrix $D=\left[D_{ij}\right]\in R^{N_{d}\times N_{d}}$ represents the disease network, with $D_{ij}$ denoting the semantic similarity of diseases $d_{i}$ and $d_{j}$. The attribute vector of $d_{i}$ is denoted as $D_{i}$.

#### 3.2.4. Drug–Disease Heterogeneous Network

Connecting edges were added to link the nodes among the three drug networks and a disease network using existing drug–disease association data (Figure 4). Let the association matrix $A\in R^{N_{r}\times N_{d}}$ denote the association between drugs and diseases, and let $A_{ij}=1$ if edges connected between $r_{i}$ and $d_{j}$ exist and $A_{ij}=0$ if no connection exists.

The matrix $U^{1}=\left[\begin{array}{cc} R^{c} & A\\ A^{T} & D \end{array}\right]\in R^{\left(N_{r}+N_{d}\right)\times \left(N_{r}+N_{d}\right)}$, which is derived from the first drug similarity, drug–disease association, and disease semantic similarity, represents the first drug–disease heterogeneous network.

Similarly, regarding the second and third drug similarities, the second and third drug–disease heterogeneous network can be generated. These two heterogeneous networks can be represented by $U^{2}=\left[\begin{array}{cc} R^{p} & A\\ A^{T} & D \end{array}\right]\in R^{\left(N_{r}+N_{d}\right)\times \left(N_{r}+N_{d}\right)}$ and $U^{3}=\left[\begin{array}{cc} R^{g} & A\\ A^{T} & D \end{array}\right]\in R^{\left(N_{r}+N_{d}\right)\times \left(N_{r}+N_{d}\right)}$.

We denote these three drug–disease heterogeneous networks by $U^{m}$, where $m\in \left\{1,\;2,\;3\right\}$.

### 3.3. Neighborhood Topology Encoding

#### 3.3.1. Multi-Scale Meta-Path Sets

The meta-path [43] can be expressed as a path shaped as $G_{1}\frac{{\;\;\;R}_{1}\;\;}{\;}G_{2}\frac{{\;\;\;R}_{2}\;\;}{\;}\cdots \frac{{\;\;\;R}_{t}\;\;}{\;}G_{t}$ (abbreviated as $G_{1}G_{2}\cdots G_{t}$). The complex relationship of node types $G_{1}$ and $G_{t}$ is described by $R=R_{1}\circ R_{2}\circ \cdots \circ R_{t}$. Two nodes can be connected to each other via different meta-paths in a heterogeneous drug–disease network. Figure 1 shows the manner by which drugs $r_{1}$ and $r_{4}$ can be connected by meta-paths r−r−r and r−d−r, with different meta-paths showing different semantics. For example, in $r_{1}-r_{2}-r_{4}$(*rrr*), drugs $r_{1}$ and $r_{4}$ may be similar if both have functions similar to $r_{2}$. In $r_{1}-d_{5}-r_{4}$(rdr), an association is indicated between both drugs and $d_{5}$, suggesting that $r_{1}$ may be similar to $r_{4}$.

Based on the structural information from $U^{m}$, we can obtain the first-order meta-paths of drug nodes with r−r and r−d to form the set $P_{r}^{\left(1\right)}=\left\{rr,rd\right\}$ of the first-order meta-paths of the drug nodes. Similarly, the second-order meta-paths of the drug nodes include r−r−r, r−d−r, r−r−d, and r−d−d, which form set $P_{r}^{\left(2\right)}=\left\{rrr,rdr,rrd,rdd\right\}$ of the second-order meta-paths of the drug node. Finally, we obtain set $P_{r}^{\left(k\right)}$($P_{d}^{\left(k\right)}$), k=1,2,…,K of the multi-scale meta-paths of the drug (disease) nodes.

#### 3.3.2. Neighbor Sets Based on Meta-Paths at Different Scales

For node $r_{i}$($d_{j}$) and the set of meta-paths $P_{r}^{\left(k\right)}$($P_{d}^{\left(k\right)}$), we can capture the drug nodes or disease nodes connected to $r_{i}$($d_{j}$) based on meta-paths of different scales. This results in a set of drug neighbor nodes ${N_{R}}_{r_{i}}^{\left(k\right)}\left({N_{R}}_{d_{j}}^{\left(k\right)}\right)$ and the disease neighbor node set ${N_{D}}_{r_{i}}^{\left(k\right)}\left({N_{D}}_{d_{j}}^{\left(k\right)}\right)$ at different scales of $r_{i}$($d_{j}$), where the first-order neighbors of the node include itself.

For the drug (disease)-type neighbors of $r_{i}$($d_{j}$), we calculated the top-$N_{k}$ neighbors that were the most similar to $r_{i}$($d_{j}$) based on their similarity to all other drugs (diseases). For the disease (drug)-type neighbors of $r_{i}$($d_{j}$), the disease (drug) nodes associated with $r_{i}$($d_{j}$) were ranked based on their occurrence frequency, and the top-$N_{k}$ nodes of the ranking were retained as neighbors of $r_{i}$($d_{j}$).

As shown in Figure 3, for $r_{1}$ and the set of meta-paths $P_{r}^{\left(1\right)}$ and $P_{r}^{\left(2\right)}$, assuming $N_{k}$ = 3, we can obtain the first-order drug neighbor nodes of $r_{1}$ based on $P_{r}^{\left(1\right)}$ via meta-paths r−r, retain the three top-ranked neighbors of $r_{1}$, and obtain the set ${N_{R}}_{r_{1}}^{\left(1\right)}=\left\{r_{1},r_{2},r_{4}\right\}$. Similarly, $r_{1}$ captures and retains the top-$N_{k}$ disease neighbors via meta-paths r−r−d and r−d−d in $P_{r}^{\left(2\right)}$, thereby forming its second-order disease neighbor set ${N_{D}}_{r_{1}}^{\left(2\right)}=\left\{d_{2},d_{5},d_{6}\right\}$.

#### 3.3.3. Aggregation of Multi-Scale Neighbor Features

We propose a fully connected neural network with mean aggregation [44] to effectively combine the network topology in $U^{m}$ with the characteristics of same-type nodes to learn the low-dimensional features of same-type neighbors at different scales. Because the learning frameworks of both drug and disease nodes are similar, we describe $r_{i}$ and its drug (disease)-type neighbors as an example.

For the *k*th-order drug neighbor set ${N_{R}}_{r_{i}}^{\left(k\right)}$ of $r_{i}$, the attribute vector $f_{r_{n}}$ of its neighbor node $r_{n}\in {N_{R}}_{r_{i}}^{\left(k\right)}$ can be obtained from the drug attribute matrix ($T^{c}$, $T^{p}$, $T^{g}$) corresponding to $U^{m}$. Because $f_{r_{n}}$ is high-dimensional and sparse, we first performed the mean aggregation of the attribute vectors of the *k*th-order drug neighbors of $r_{i}$, and the aggregated vector ${h_{R}}_{r_{i}}^{\left(k\right)}$ is expressed as:

(3) $${h_{R}}_{r_{i}}^{\left(k\right)}=\;mean\left(\left\{{f_{r_{i}},\ldots ,f}_{r_{n}},\ldots \right\}\right),r_{n}\in {N_{R}}_{r_{i}}^{\left(k\right)}$$

Subsequently, we project ${h_{R}}_{r_{i}}^{\left(k\right)}$ into the low-dimensional feature space through a fully connected network and obtain the low-dimensional *k*th-order drug neighbor feature vector ${u_{R}}_{r_{i}}^{\left(k\right)}$ as follows:

(4) $${u_{R}}_{r_{i}}^{\left(k\right)}=\sigma \left(W_{R}^{\left(k\right)}{h_{R}}_{r_{i}}^{\left(k\right)}+b_{R}^{\left(k\right)}\right)$$

where σ denotes the activation function ReLU [45], $W_{R}^{\left(k\right)}$ the weight matrix when the neighbor type is a drug, and $b_{R}^{\left(k\right)}$ the bias vector. *K* denotes the total number of orders, and K=2 in our model.

#### 3.3.4. Same-Type Neighbor Topology Encoding Based on Neighbor-Scale-Level Attention

Because the drug (disease)-type neighbor node information at different scales of $r_{i}$ contributes differently to the learning of the drug (disease) neighbor topological representation of $r_{i}$, we established a neighbor-scale-level attention to learn the attention weights of order 1-*k* neighbor feature vectors of the same type. For the *k*th-order drug neighbor feature ${u_{R}}_{r_{i}}^{\left(k\right)}$ of $r_{i}$, with attention score $s_{k}^{Scale}$,

(5) $${s_{k}^{Scale}=h}^{Scale}\tanh\left(W^{Scale}{u_{R}}_{r_{i}}^{\left(k\right)}+b^{Scale}\right),$$

where $h^{Scale}$ is the weight vector at the neighbor scale level; $W^{Scale}$ and $b^{Scale}$ are the weight matrix and bias vector, respectively. The normalized attention coefficient is $\alpha_{k}^{Scale}$, which can be obtained using the softmax function, as follows:

(6) $$\alpha_{k}^{Scale}=\frac{exp\left(s_{k}^{Scale}\right)}{\sum_{n\in K}exp\left(s_{n}^{Scale}\right)}$$

The drug neighbor topology representation ${u_{R}}_{r_{i}}$ of $r_{i}$ obtained using the attention mechanism is:

(7) $${u_{R}}_{r_{i}}=\sum_{k}\alpha_{k}^{Scale}{u_{R}}_{r_{i}}^{\left(k\right)}$$

#### 3.3.5. Neighbor Topology Encoding Based on Attention Enhancement at the Neighbor Topology Level

$r_{i}$ contains two types of neighbor nodes, drug and disease, whose neighbor topologies are represented as ${u_{R}}_{r_{i}}$ and ${u_{D}}_{r_{i}}$, respectively. However, the importance of different types of neighbor nodes for association prediction varies, and neighbor-topology-level attention is proposed to enhance the neighbor topology representation of $r_{i}$. The attention score for the same-type neighbor topology representation of $r_{i}$ is:

(8) $${s_{t}^{Topo}=h}^{Topo}\tanh\left(W^{Topo}{u_{t}}_{r_{i}}+b^{Topo}\right),$$

where $t\in \left\{R,D\right\}$, $W^{Topo}$ and $h^{Topo}$ are the neighbor-topology-level weight matrix and weight vector, respectively, and $b^{Topo}$ is a bias vector. The normalized attention weights $\alpha_{t}^{Topo}$ are expressed as follows:

(9) $$\alpha_{t}^{Topo}=\frac{exp\left(s_{t}^{Topo}\right)}{\sum_{n\in 2}exp\left(s_{n}^{Topo}\right)}$$

Finally, the augmented representation of the $r_{i}$ neighbor topology obtained using the attention mechanism is $u_{r_{i}}$, expressed as follows:

(10) $$u_{r_{i}}=\sum_{t}\alpha_{t}^{Topo}{u_{t}}_{r_{i}}$$

Here, $u_{r_{i}}^{\left(m\right)}$ denotes the neighboring topological representation obtained by $r_{i}$ in $U^{m}$, where $m\in \left\{1,2,3\right\}$.

Similarly, the neighbor topology representation $u_{d_{j}}^{\left(m\right)}$ of $d_{j}$ in $U^{m}$ can be obtained. These neighboring topological representations are used to form the feature matrices *S* of $r_{i}$–$d_{j}$ node pairs, as follows:

(11) $$S=\left[\begin{array}{ccc} u_{r_{i}}^{\left(1\right)} & u_{r_{i}}^{\left(2\right)} & u_{r_{i}}^{\left(3\right)}\\ u_{d_{j}}^{\left(1\right)} & u_{d_{j}}^{\left(2\right)} & u_{d_{j}}^{\left(3\right)} \end{array}\right]\in R^{2\times \left(N_{f}+N_{f}+N_{f}\right)},$$

where $N_{f}$ denotes the dimension number of the neighbor topology representation.

#### 3.3.6. CNN-Based Pairwise Neighbor Topology Encoding

The feature matrix of the first branch *S* is passed into the CNN, which learns the $r_{i}$–$d_{j}$ neighbor topology representations. We filled the periphery of *S* with zeros to learn the edge features of *S* and then obtained the new matrix $\hat{S}$.

We established a CNN module using convolutional and pooling layers. The filter length and breadth relative to the convolution layer are denoted by $w_{l}$ and $w_{h}$, respectively; a total of $n_{conv}$ filters were used. After applying the convolution filter $W_{conv}\in R^{w_{l}\times w_{h}\times n_{conv}}$ to $\hat{S}$, a feature map $Z\in R^{n_{conv}\times \left(4-w_{l}+1\right)\times \left(2+N_{f}+N_{f}+N_{f}-w_{h}+1\right)}$ was generated. ${\hat{S}}_{k,i,j}$ represents the sliding of the *k*-th filter to position (i,j) of $\hat{S}$, and it is defined as:

(12) $${\hat{S}}_{k,i,j}=\hat{S}\left(i:i+w_{l},j:j+w_{h}\right),\;{\hat{S}}_{k,i,j}\in R^{w_{l}\times w_{h}},$$

where $i\in \left[1,4-w_{l}+1\right]$, $j\in \left[1,2+\left(N_{f}+N_{f}+N_{f}\right)-w_{h}+1\right]$, and $k\in \left[1,n_{conv}\right]$. The element value $Z_{k}\left(i,j\right)$ of the filter $W_{k,i,j}$ sliding on ${\hat{S}}_{k,i,j}$ to $Z_{k}$ is:

(13) $$Z_{k}\left(i,j\right)=\sigma \left(W_{k,i,j}*{\hat{S}}_{k,i,j}+b\left(k\right)\right),$$

where σ is the ReLU function and b the bias vector. The position (i,j) in the feature map $Z_{k}$ is represented by $Z_{k}\left(i,j\right)$.

The more significant features of $Z_{k}$ were extracted using the max-pooling layer. The filter length of the max-pooling layer is $w_{e}$, and the width is $w_{b}$. The *k*-th feature map of all feature maps *P* output by the pooling layer is $P_{k}$, and $P_{k}\left(i,j\right)$ can be calculated as:

(14) $$P_{k}\left(i,j\right)=Max\left(Z_{k}\left(i:i+w_{e},j:j+w_{b}\right)\right),$$

where $i\in \left[1,5-w_{l}-w_{e}+1\right]$, $j\in [1,\left(N_{f}+N_{f}+N_{f}\right)+3-w_{h}-w_{b}+1]$, and $k\in \left[1,n_{conv}\right]$.

In the CNN module, we set the number of filters in the convolutional layer to 16, the kernel size to 2 × 2, and the stride size to 1. In the pooling layer, the kernel size was set to 2 × 2, and the step size and zero-padding were set to 1 and 0, respectively. After performing processing in the convolution and max-pooling layers, the output vector $z_{NT}$ was obtained. Subsequently, $z_{NT}$ was input to the fully connected and softmax layer [46], which yielded the association probability distributed for the first branch, as follows:

(15) $${Score}_{NT}=softmax\left(W_{soft1}z_{NT}+b_{soft1}\right),$$

where $W_{soft1}$ is the first branch of the fully connected layer’s weight matrix and $b_{soft1}$ is the corresponding bias vector. ${Score}_{NT}$ indicates the association probability distribution for the C(C=2) classification, including the likelihood of a drug and disease being associated and otherwise.

### 3.4. Encoding Pairwise Node Attributes

#### 3.4.1. Attribute Embedding Matrix for Drug–Disease Pairs

**Figure 5:**
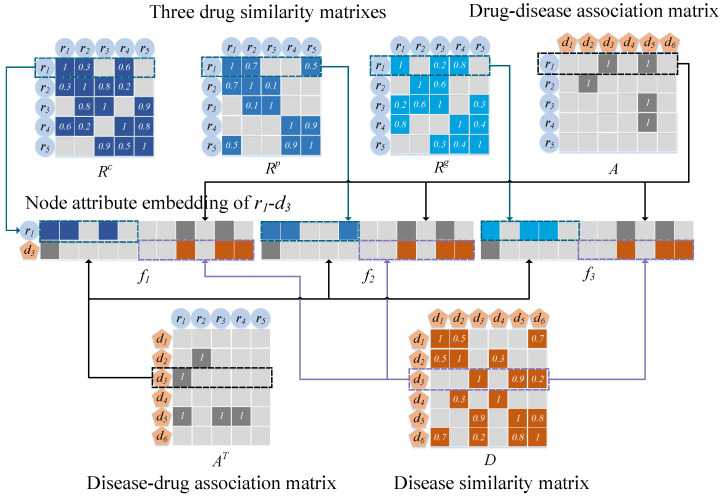
Illustration of constructing an attribute embedding matrix for a pair of drug and disease nodes.

We introduced an embedding strategy to extract the nodal attributes of drug–disease pairs (Figure 5). If $r_{i}$($d_{j}$) is similar (related) to a more typical drug or related (similar) to a disease, then $r_{i}$–$d_{j}$ is likely to be related. Therefore, information regarding the properties of drugs and diseases must be learned from the pairwise node level.

For a heterogeneous drug–disease network $U^{m}$, $U_{i}^{m}$ contains the *m*-th similarity of $r_{i}$ with all drugs and the association with all diseases, and $U_{N_{r}+j}^{m}$ contains the association of $d_{j}$ with all drugs and the similarity with all diseases. Therefore, we used the attribute vectors $U_{i}^{m}$ and $U_{N_{r}+j}^{m}\left(m=1,2,3\right)$ to perform splicing such that the attribute embedding matrix *P* of $r_{i}$ and $d_{j}$ can be obtained. *P* is expressed as follows:

(16) $$P=\left[\begin{array}{ccc} U_{i}^{1} & U_{i}^{2} & U_{i}^{3}\\ U_{N_{r}+j}^{1} & U_{N_{r}+j}^{2} & U_{N_{r}+j}^{3} \end{array}\right]\in R^{2\times \left(\left(N_{r}+N_{d}\right)\times 3\right)},$$

where *P* has a dimension of $2\times \left(\left(N_{r}+N_{d}\right)\times 3\right)$.

#### 3.4.2. CAE-Based Pairwise Node Attribute Encoding

Because the node attribute matrix *P* obtained from the three heterogeneous networks is high-dimensional and sparse, meaningless and non-representative information may be present. Therefore, we performed encoding and decoding based on a CAE to comprehensively learn the attribute information of drug–disease pairs in the original data distribution, as shown in Figure 3.

**Encoder**: Two hidden layers, each comprising a convolutional layer and a max-pooling layer, constitute the encoder. The edge features of *P* should be preserved and learned via zero-padding. The first hidden layer uses the zero-padded *P* as input and yields the feature map $Z_{Encoder}^{\left(1\right)}$ encoded as:

(17) $$Z_{Encoder}^{\left(1\right)}=max\left(\sigma \left(W_{Encoder}^{\left(1\right)}*P+b_{Encoder}^{\left(1\right)}\right)\right)$$

Subsequently, the feature map of the *t*-th layer $Z_{Encoder}^{\left(t\right)}$ is generated as follows:

(18) $$Z_{Encoder}^{\left(t\right)}=max\left(\sigma \left(W_{Encoder}^{\left(t\right)}*Z_{Encoder}^{\left(t-1\right)}+b_{Encoder}^{\left(t\right)}\right)\right)$$

where σ is the ReLU function. $W_{Encoder}^{\left(t\right)}$ denotes the encoder’s *t*-th hidden layer’s weight matrix, and $b_{Encoder}^{\left(t\right)}$ is the corresponding bias vector. $t=2,\ldots ,L_{En}$. $L_{En}$ indicates the encoder’s total number of layers, and the convolution computation is indicated by “*”; max denotes the max-pooling processing for capturing the most critical features within every feature map by downsampling the potential representations acquired from the convolution layer.

**Decoder**: Using the decoder, we projected the $Z_{Encoder}^{\left(L_{En}\right)}$ code such that it returns to its initial space and reassembled it to obtain the decoding matrix. The variance between the decoding matrix and the initial matrix *P* was evaluated, and an optimal coded feature map was obtained. Three hidden layers, each with a transposed convolutional layer, constitute the decoder. For $Z_{Encoder}^{\left(L_{En}\right)}$ as the input of the first hidden layer of the decoder, the feature map $Z_{Decoder}^{\left(1\right)}$ is obtained as follows:

(19) $$Z_{Decoder}^{\left(1\right)}=\sigma \left(W_{Decoder}^{\left(1\right)}{⋆Z}_{Encoder}^{\left(L_{En}\right)}+b_{Decoder}^{\left(1\right)}\right)$$

(20) $$Z_{Decoder}^{\left(l\right)}=\sigma \left(W_{Decoder}^{\left(l\right)}⋆Z_{Decoder}^{\left(l-1\right)}+b_{Decoder}^{\left(l\right)}\right)$$

where $W_{Decoder}^{\left(l\right)}$ is the weight matrix of the decoder and $b_{Decoder}^{\left(l\right)}$ is the decoder’s bias vector. $l=2,\ldots ,L_{De}$. A total of $L_{De}$ decoder layers are involved. The operator “⋆” indicates the transposed convolution computation. The reconstructed matrix $\hat{P}$ is the output $Z_{Decoder}^{\left(L_{De}\right)}$ of the last layer of the decoder.

**Optimization**: Our optimization objective was to render $\hat{P}$ as consistent as possible with the input *P*. The loss function is expressed as:

(21) $${loss}_{auto}=\frac{1}{T_{train}}\sum_{n=1}^{T_{train}}{\left(P_{n}-{\hat{P}}_{n}\right)}^{2},$$

where *P* is the input of the encoder, $\hat{P}$ the output at the decoder, $T_{train}$ the number of training samples, and $P_{n}$ the embedding matrix of the *n*th drug–disease pair in the corresponding training sample. Adam’s algorithm [47] was used to optimize ${loss}_{auto}$. The back propagation [48] approach was used to train the CAE and update ${loss}_{auto}$. Using the iterative algorithm, the pairwise property encoding was regarded as the output $Z_{Encoder}^{\left(L_{En}\right)}$ of the last encoder layer, denoted by $F_{PA}$.

To acquire the association probability of the second branch of node pair $r_{i}$–$d_{j}$${Score}_{PA}$, $F_{PA}$ was processed in the fully connected and softmax layer. ${Score}_{PA}$ is expressed as:

(22) $${Score}_{PA}=softmax\left(W_{soft2}F_{PA}+b_{soft2}\right),$$

where $W_{soft2}$ and $b_{soft2}$ are the weight matrix and bias vector of the fully connected second branch, respectively. ${Score}_{PA}$ is the association probability distribution for the C(C=2) classification.

### 3.5. Final Integration and Optimization

The loss function in the first branch can be expressed as the cross-entropy between the true label $y_{NT}$ and the drug–disease association prediction result ${Score}_{NT}$, as follows:

(23) $${loss}_{NT}=-\sum_{i=1}^{N_{train}}\sum_{j=1}^{C}{y_{NT}}_{j}\log\left({{Score}_{NT}}_{j}\right),$$

where $N_{train}$ is the set of training samples and ${y_{NT}}_{j}$ represents the probability of a drug–disease association. If an $r_{i}$–$d_{j}$ pair has an association, then ${y_{NT}}_{j}$ is 1; otherwise, it is 0. In the second branch, the cross-entropy loss function ${loss}_{PA}$ is defined as:

(24) $${loss}_{PA}=-\sum_{i=1}^{N_{train}}\sum_{j=1}^{C}{y_{PA}}_{j}\log\left({{Score}_{PA}}_{j}\right)$$

We trained the loss functions ${loss}_{NT}$ and ${loss}_{PA}$ separately until their minimum values were attained. The final correlation prediction score is calculated as follows:

(25) $$Score=\lambda \times {Score}_{NT}+\left(1-\lambda \right)\times {Score}_{PA},$$

where λ denotes a hyperparameter that ranges from 0 to 1 and was used to measure the contribution of neighboring topologies and pairwise node attributes to the association prediction score.

## 4. Conclusions

We proposed the NAPred method to determine the association between drug candidates and diseases. The three proposed heterogeneous networks facilitated neighbor topology extraction and pairwise node attribute embedding using multiscale meta-paths. A framework comprising a convolutional neural network with attention mechanisms and CAE was constructed to encode and integrate neighbor topological representations and pairwise attribute representations. Two attention mechanisms were proposed to assign greater weights to multi-scale features and topologies. NAPred’s ability to discover potentially relevant diseases for drugs was validated through case studies and a cross-validation of five drugs. Numerous experimental results showed that NAPred’s predictions outperformed existing methods. Our predictive model serves as a tool for screening to recognize potential drug–disease associations, thereby allowing biologists to conduct wet laboratory research for determining real drug–disease associations.

## Figures and Tables

**Table 1:** The statistical results of the paired Wilcoxon test on the AUCs over all the 763 drugs by comparing NAPred and all other five methods.

	GFPred	CBPred	SCMFDD	LRSSL	MBiRW	HGBI
*p*-value of AUC	5.27051 × 10${}^{-25}$	1.83480 × 10${}^{-33}$	5.49787 × 10${}^{-65}$	5.31080 × 10${}^{-47}$	2.89205 × 10${}^{-62}$	1.74747 × 10${}^{-81}$
*p*-value of AUCPR	3.42304 × 10${}^{-31}$	4.72506 × 10${}^{-47}$	1.81013 × 10${}^{-71}$	8.63715 × 10${}^{-65}$	4.68094 × 10${}^{-59}$	4.85712 × 10${}^{-89}$

**Table 2:** The top-10 candidate diseases of 5 drugs.

Drug Name	Rank	Disease Name	Description	Rank	Disease Name	Description
	1	Staphylococcal Infections	CTD, PubChem	6	Staphylococcal Skin	PubChem
					Infections	
	2	Pneumonia, Bacterial	ClinicalTrials	7	Streptococcal Infections	CTD, ClinicalTrials
Ampicillin	3	Urinary Tract Infections	CTD, DrugBank,	8	Osteomyelitis	PubChem,
			PubChem			ClinicalTrials
	4	Wound Infection	PubChem, ClinicalTrials	9	Postoperative Complications	PubChem
	5	Proteus Infections	Inferred Candidate	10	Bacterial Infections	CTD, DrugBank,
			by 2 Literature Works			ClinicalTrials
	1	Escherichia coli Infections	CTD, PubChem, ClinicalTrials	6	Salmonella Infections	DrugBank, PubChem, ClinicalTrials
	2	Urinary Tract Infections	DrugBank, PubChem,	7	Enterobacteriaceae Infections	PubChem, ClinicalTrials
			ClinicalTrials			
Ceftriaxone	3	Haemophilus Infections	PubChem	8	Septicemia	DrugBank, PubChem,
						ClinicalTrials
	4	Gonorrhea	DrugBank, PubChem,	9	Endocarditis, Bacterial	DrugBank, ClinicalTrials
			ClinicalTrials			
	5	Gram-Negative Bacterial	Inferred Candidate	10	Pseudomonas Infections	PubChem
		Infections	by 1 Literature Work			
	1	Urinary Tract Infections	CTD, PubChem	6	Leukemia, Lymphoid	CTD, DrugBank,
						ClinicalTrials
	2	Leukemia, Myeloid,	CTD, DrugBank,	7	Bronchitis	CTD
		Acute	ClinicalTrials			
Doxorubicin	3	Escherichia coli Infections	CTD	8	Sarcoma	CTD, DrugBank,
						ClinicalTrials
	4	Neoplasms	ClinicalTrials, PubChem	9	Gonorrhea	Unconfirmed
	5	Staphylococcal Infections	CTD, PubChem	10	Precursor Cell Lymphoblastic	CTD
					Leukemia-Lymphoma	
	1	Gonorrhea	DrugBank, PubChem	6	Gram-Positive Bacterial Infections	PubChem
	2	Gram-Negative Bacterial	PubChem	7	Staphylococcal Infections	CTD, DrugBank,
Erythromycin		Infections				PubChem
	3	Chancroid	DrugBank, PubChem	8	Pneumonia, Mycoplasma	Unconfirmed
	4	Bacterial Infections	DrugBank, PubChem	9	Neurosyphilis	PubChem
	5	Neisseriaceae Infections	DrugBank	10	Chlamydiaceae Infections	DrugBank, ClinicalTrials
	1	Candidiasis, Cutaneous	DrugBank, PubChem,	6	Tinea Capitis	DrugBank, PubChem
			ClinicalTrials			
	2	Tinea Versicolor	DrugBank, PubChem,	7	Fungemia	DrugBank, PubChem,
			ClinicalTrials			ClinicalTrials
Itraconazole	3	Tinea Pedis	DrugBank, PubChem	8	Skin Diseases, Infectious	PubChem, ClinicalTrials
	4	Leishmaniasis	CTD, PubChem,	9	AIDS-Related Opportunistic	ClinicalTrials
			ClinicalTrials		Infections	
	5	Chromoblastomycosis	DrugBank, PubChem	10	Candidiasis	CTD, DrugBank, PubChem

## Supplementary Materials

The following are available at: https://www.mdpi.com/article/10.3390/ijms23073870/s1.
