# Supplementary material for: Integration of Neighbor Topologies Based on Meta-Paths and Node Attributes for Predicting Drug-Related Diseases
Source: Int J Mol Sci. 2022 Mar 31;23(7):3870. doi: 10.3390/ijms23073870 (PMC8999005; doi:10.3390/ijms23073870)
Supplement: Supplementary file 1 [file ijms-23-03870-s001.zip › Table S1.pdf]

Supplementary Table S1. Average AUC and AUPR for different folds of cross-validations.

	5-fold cross-validation	10-fold cross-validation
Average AUC	0.978	0.986
Average AUPR	0.391	0.404
