# Supplementary material for: Integration of Neighbor Topologies Based on Meta-Paths and Node Attributes for Predicting Drug-Related Diseases
Source: Int J Mol Sci. 2022 Mar 31;23(7):3870. doi: 10.3390/ijms23073870 (PMC8999005; doi:10.3390/ijms23073870)
Supplement: Supplementary file 1 [file ijms-23-03870-s001.zip › Table S2.pdf]

Supplementary Table S2. Top 10 drug candidates related to betamethasone, acetaminophen, etoposide, flurbiprofen and verapamil.

Drug Name	Rank	Disease name	Description	Rank	Disease name	Description
betamethasone	1	Inflammation	CTD, DrugBank, PubChem, ClinicalTrials	6	Psoriasis	CTD, DrugBank, PubChem, ClinicalTrials
	2	Scalp Dermatoses	DrugBank, PubChem, ClinicalTrials	7	Pruritus	CTD, DrugBank, PubChem
	3	Foot Dermatoses	DrugBank	8	Skin Diseases, Eczematous	CTD, DrugBank, PubChem, ClinicalTrials
	4	Facial Dermatoses	CTD, DrugBank, PubChem	9	Acne Vulgaris	CTD
	5	Skin Diseases	CTD, DrugBank, PubChem	10	Dermatitis	CTD, DrugBank, PubChem, ClinicalTrials
acetaminophen	1	Osteoarthritis	CTD, DrugBank, PubChem, ClinicalTrials	6	Arthritis, Juvenile Rheumatoid	CTD, PubChem
	2	Fever	CTD, DrugBank, PubChem, ClinicalTrials	7	Spondylitis, Ankylosing	CTD, DrugBank, PubChem
	3	Arthritis, Rheumatoid	CTD, DrugBank, PubChem, ClinicalTrials	8	Pain, Postoperative	CTD, DrugBank, PubChem, ClinicalTrials
	4	Inflammation	CTD, PubChem	9	Gout	CTD, PubChem
	5	Dysmenorrhea	CTD, DrugBank, PubChem	10	Asthma	CTD
etoposide	1	Urinary Tract Infections	CTD	6	Trophoblastic Neoplasms	DrugBank, PubChem
	2	Lymphoma	CTD, DrugBank, PubChem, ClinicalTrials	7	Sarcoma	CTD, DrugBank, PubChem, ClinicalTrials
	3	Bronchitis	CTD	8	Precursor Cell Lymphoblastic Leukemia-Lymphoma	CTD
	4	Leukemia, Lymphoid	CTD, DrugBank, PubChem, ClinicalTrials			
	5	Breast Neoplasms	CTD, PubChem, ClinicalTrials	9	Neoplasms	CTD, DrugBank, PubChem, ClinicalTrials
				10	Escherichia coli Infections	CTD
flurbiprofen	1	Pain	CTD, DrugBank, PubChem, ClinicalTrials	6	Gout	CTD, PubChem
	2	Arthritis, Juvenile Rheumatoid	CTD, DrugBank, PubChem	7	Photophobia	PubChem
				8	Pain, Postoperative	CTD, DrugBank,

	3	Dysmenorrhea	DrugBank, PubChem			PubChem, ClinicalTrials
	4	Fever	CTD, PubChem	9	Bursitis	PubChem
	5	Spondylitis, Ankylosing	CTD, DrugBank, PubChem	10	Arthritis	CTD, PubChem, ClinicalTrials
verapamil	1	Atrial Fibrillation	CTD, PubChem, ClinicalTrials	6	Pain	CTD, PubChem
	2	Hypertension	CTD, DrugBank, PubChem, ClinicalTrials	7	Angina Pectoris	CTD, DrugBank, PubChem
	3	Tachycardia, Supraventricular	DrugBank, PubChem, ClinicalTrials	8	Arrhythmias, Cardiac	CTD, DrugBank, PubChem
	4	Angina Pectoris, Variant	unconfirmed	9	Asthma	CTD, PubChem
	5	Ventricular Fibrillation	CTD, PubChem	10	Schizophrenia	CTD, PubChem
